# Assessment of chemotherapy‐induced neurotoxicity using a point‐of‐care nerve conduction study device

**DOI:** 10.1002/cnr2.1677

**Published:** 2022-07-11

**Authors:** Anna Jokimäki, Sanna Iivanainen, Raija Mikkonen, Kallio Mika, Jussi Koivunen

**Affiliations:** ^1^ Department of Oncology and Radiotherapy Oulu University Hospital Oulu Finland; ^2^ Institute of Clinical Medicine, Faculty of Health Medicine University of Eastern Finland Kuopio Finland; ^3^ Medical Research Center, Oulu University Hospital and Cancer and Translational Research Unit University of Oulu Oulu Finland; ^4^ Department of Clinical Neurophysiology Oulu University Hospital Oulu Finland

**Keywords:** chemotherapy, neuropathy, point‐of‐care device

## Abstract

**Background:**

Management for chemotherapy‐induced peripheral neuropathy (CIPN) includes prompt recognition and dose reduction or discontinuation of the neurotoxic agents. CIPN remains under‐detected in routine clinical practice and better methods for its early detection are warranted.

**Aims:**

To evaluate the feasibility of a point‐of‐care device in the early detection of CIPN.

**Methods and Results:**

Cancer patients (*n* = 12) scheduled to receive neurotoxic chemotherapy docetaxel, oxaliplatin (OX), or vincristine were recruited for the pilot study (NCT04778878). The patients were assessed with a point‐of‐care nerve conduction study device (Mediracer® NCS), EORTC QLQ‐CIPN20 and NPSI questionnaires, and healthcare professional‐assessed CTCAE‐based grading at baseline and thereafter every 6‐weeks up to 18 weeks or until chemotherapy discontinuation. The set‐up of point‐of‐care device was easy but it only provide successful NCS measurement results in 55% of the patients. The factors related to failed measurement were older age, more frequent comorbidities, and a history of smoking. With the follow‐up measurements, decreasing median nerve mean conduction velocity and amplitude, and increasing median nerve mean distal latency were detected on OX‐patients. Of the used questionnaires, NPSI was found to be non‐feasible with majority of the patients failing to complete the questionnaire while CIPN20 was feasible on all the subjects. CIPN20 score did not show any changes in the follow‐up.

**Conclusions:**

Point‐of‐care assessment for NCS was feasible but measurements frequently failed especially on patients with pre‐existing high‐risk for neuropathy. OX‐treated showed decreasing NCS results while other measures were unable to access the change. The system should be further validated with a larger patient cohort preferably treated with OX and low‐risk for pre‐existing neuropathy.

## 1 INTRODUCTION

Peripheral neuropathy is a common side effect of chemotherapy and it concerns a large amount of patients worldwide. Chemotherapy‐induced peripheral neuropathy (CIPN) may result in persistent symptoms leading to the deterioration of quality of life (QoL) in cancer survivors. In a systematic review and meta‐analysis consisting 4179 adult cancer patients, CIPN affected 60.0% of patients at 3 months, and 30.0% at 6 months or more.^1^ CIPN may potentially cause long‐term effects on activities of daily living (ADL),^2^ concerning 47% of patients after years since the end of treatment.

Neurotoxic chemotherapeutic agents are widely used in the treatment of various cancers. As an example, vincristine, oxaliplatin, and docetaxel, are common neurotoxic agents used in the treatment of malignancies such as aggressive lymphomas, colorectal, and prostate cancers.^3^, ^4^, ^5^, ^6^, ^7^, ^8^ Mechanism, timing, and severity of the chemotherapy‐induced neuropathy may vary between different agents.

International guidelines for systemic anticancer therapy‐induced neurotoxicity sums up the evidence‐based measures for detection, prevention, and management of CIPN.^9^ CIPN commonly presents as sensory axonal neuropathy, but also motor and autonomic functions may be affected. There are no effective pharmacologic agents or strategies for CIPN prevention. Therefore, prompt recognition of CIPN and subsequent dose reduction or discontinuation of the neurotoxic agent is recommended to prevent potential worsening of the condition.

In clinical practice, significant underdetection and underrating of CIPN is common. Major discordance between patient‐ and physician‐assessed neuropathy grading has previously been shown.^10^, ^11^ Invasive neurophysiological methods to detect CIPN are non‐feasible to be applied to routine clinical practice, and there is an unmet need for point‐of‐care, quantitative screening procedures for CIPN. Some previous studies have investigated NCS in the detection of CIPN. Sural SNAP (sensory nerve action potential) and SNCV (sensory nerve conduction velocity) measured by a point‐of‐care nerve conduction device (POCD) were found to correlate with CIPN severity in Japanese patients receiving either platinum compounds, taxanes, vincristine, or bortezomib.^12^ Sudomotor function assessed by Sudoscan was found to correlate with the total neuropathy score clinical version (TNSc) in a preliminary study of patients receiving neurotoxic chemotherapy. Sharma and al. tested the LDI‐FLARE technique in patients with established CIPN and matched healthy controls, and found that LDI‐FLARE was useful in detecting CIPN compared with other neurophysiological examinations (SNAP, SNCV, and vibration perception threshold).^13^ Semmes‐Weinstein monofilaments have also been studied in this purpose.^14^ However, none of these objective measurement devices/means are recommended by the international guidelines, and nerve conduction measurements often do not mirror the severity of CIPN.^9^

The purpose of this pilot study is to evaluate the feasibility of a point‐of‐care NCS (Mediracer® NCS) in the early detection of CIPN in patients receiving neurotoxic agents (docetaxel, oxaliplatin or vincristine) as a part of their chemotherapy regimen, and to compare the NCS results with EORTC QLQ‐CIPN20 and NPSI questionnaires, and healthcare professional‐assessed CTCAE‐based grading.

## 2 METHODS

### 2.1 Patients and assessments

**FIGURE 1:**
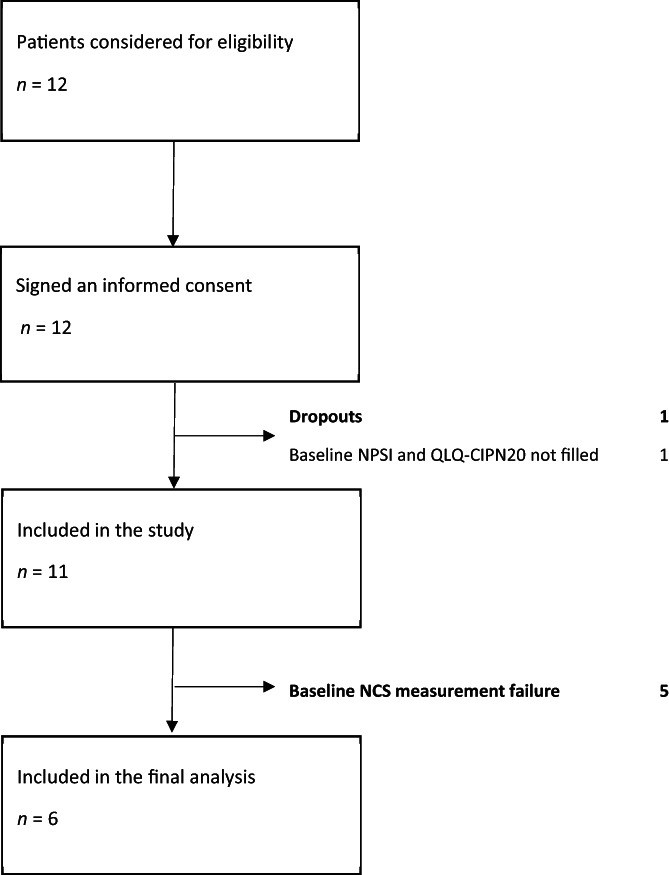
Flow chart of the patient recruitment

Twelve patients scheduled to receive a neurotoxic chemotherapy regimen (containing either docetaxel, oxaliplatin, or vincristine) at Oulu University Hospital (Oulu, Finland) were recruited to the study between October 2019 and November 2020. Major inclusion criteria were age > 18 years, ECOG 0–2, compliance with study procedures, and intention to receive neurotoxic chemotherapy (vincristine, oxaliplatin, or docetaxel). Exclusion criteria included unfit/not suitable for chemotherapy at baseline, any prior postoperative or post‐traumatic conditions affecting sensory/motoric peripheral nerves, and general vulnerability affecting the participation in the trial. Flowchart of patient accrual and analysis is shown in Figure 1.

Patients were assessed for NCS, neuropathy questionnaires, and symptom grading by NCI‐CTCAE before chemotherapy initiation, and thereafter every 6 weeks until a minimum follow‐up of 12 weeks was reached. If initial NCS failed, further follow‐up was canceled and only baseline data were included in the study analysis.

The study was approved by the ethics committee of Northern Osthrobothnia Hospital District (study n:o 48/2019) and registered in an international clinical study registry (NCT04778878). The study was carried out in accordance with the principles of the Declaration of Helsinki. All patients provided written informed consent.

### 2.2 Nerve conduction study measurements

Mediracer® NCS is CE marked and FDA‐approved a point‐of‐care noninvasive hand‐held NCS device. Formerly, it has been studied as a part of the diagnostic assessment of carpal tunnel syndrome^15^, ^16^, ^17^ The device is technically suitable for assessing peripheral sensory and motor NCS due to various conditions, including diabetic neuropathy. The feasibility of the Mediracer® NCS device in the assessment of CIPN has not yet been studied. All the study personnel was individually trained by a Mediracer to conduct the NCS measurements according to the manufacturer's references, and to minimize inter‐measurement and inter‐examiner variability of the results.

### 2.3 Neuropathy questionnaires and symptom grading

The NPSI (Neuropathic Pain Symptom Inventory) questionnaire has been developed and validated^18^ to study different symptoms of neuropathic pain. Each of the items (spontaneous ongoing or paroxysmal pain, evoked pain, meaning mechanical and thermal allodynia/hyperalgesia, and dysesthesia/paresthesia) are quantified on a (0–10) numerical scale. It allows the discrimination and quantification of different types of neuropathic pain.

The EORTC QLQ‐CIPN20 (European Organization for Research and Treatment of Cancer Chemotherapy‐Induced Peripheral Neuropathy Module) questionnaire has been developed and the sensory and motor subscales have been validated^19^, ^20^ for the use in different patient groups exposed to potentially neurotoxic drugs. It includes 20 items about CIPN‐related symptoms (sensory, motor, and autonomic domains) and functional limitations of patients.

National Cancer Institute Common Terminology Criteria for Adverse Events (NCI‐CTCAE) version 5.0 grade of CIPN (sensory and motor symptoms) is generally used as an objective measure of neuropathy symptoms. NCI‐CTCAE Grade 1 refers to asymptomatic; Grade 2: moderate symptoms limiting instrumental ADL, Grade 3: severe symptoms limiting self‐care ADL and grade 4: life‐threatening consequences.

### 2.4 Statistics

At baseline, patient demographics and ECOG performance status, planned chemotherapy regimen, prior diseases/conditions affecting the peripheral sensory/motor nerve system, and grade of peripheral neuropathy (by NCI‐CTCAE) were collected by the study physicians or study nurse. NPSI and EORTC QLQ‐CIPN20 were completed by the patient before or at the initiation of chemotherapy. Baseline NCS using Mediracer® NCS was performed by physician/study nurse before or at the initiation of chemotherapy. All the study physicians and the study nurse were individually trained by a Mediracer Ltd staff member (K.F.) to conduct the NCS according to the manufacturer's references to minimize inter‐measurement and inter‐examiner variability of the results.

The follow‐up was done at 6 weeks interval, or coinciding with the patients' scheduled treatment visits, until a minimum follow‐up of 12 weeks was reached. Follow‐up included ECOG scoring and CTCAE scoring of CIPN symptoms by the study physician or study nurse, and listing the ongoing chemotherapy treatment regimen at that time point (generic names of drugs, indication, dates of received infusions). If the chemotherapy regimen was changed or terminated earlier than expected, date and additional data on symptoms/problems leading to the change/termination were collected. NPSI and EORTC QLQ‐CIPN20 questionnaires were completed by the patient. Follow‐up NCS was done by the study physicians or the study nurse.

Study results were analyzed when the last included patient had a total of 12 weeks of follow‐up. Statistical analysis was performed using IBM SPSS Statistics for Windows, versions 25 and 27. GraphPad Prism version 5 was used for the graphic presentation of the data. Before data analysis, the NCS measurements were reviewed by a clinical neurophysiologist (M.K.).

The study was approved by the ethics committee of Northern Osthrobothnia Hospital District (48/2019) and is available in public database (NCT04778878). The study was carried out in accordance with the principles of the Declaration of Helsinki. All patients provided written informed consent.

## 3 RESULTS

### 3.1 Patient population

The patient accrual was carried out in a single study center (Oulu University Hospital, Oulu, Finland) between October 2019 and November 2020, and flowchart of accrual is presented in Figure 1. Demographics of the study population are described in detail at Table 1. In brief, the median age was 69 years, and there were more males (*n* = 8) than females (*n* = 3). Majority of the patients were scheduled to be given docetaxel at 3 weeks interval (DOC Q3W) for prostate cancer (*n* = 6), and most of them (*n* = 5) treated in the castration‐sensitive setting. Oxaliplatin (OX Q2W or Q3W) was started as a treatment for three patients, of whom two were adjuvant‐treated, and one patient had metastatic colon cancer. Vincristine (VIN Q3W) was scheduled for two patients as a part of the treatment protocol for diffuse large B‐cell lymphoma (DLBCL). Most of the patients had comorbidities (*n* = 7, 64%), a history of smoking (*n* = 6, 55%), tended to be slightly overweight (mean BMI 26.6 kg/m^2^), and had normal renal function measured by GFR (glomerular filtration rate estimated by CKD‐EPI equation).^21^

**TABLE 1:** Patient demographics

Characteristics	*n* (%)
All	11 (100)
Age (median, range)	69 (51–76)
Gender	
Female	3 (27)
Male	8 (73)
ECOG	
0	5 (46)
1	6 (55)
Neurotoxic chemotherapy	
Docetaxel	6 (55)
Oxaliplatin	3 (27)
Vincristine	2 (18)
Comorbidities	
Hypertension	4 (36)
Diabetes	2 (18)
Body mass index, kg/m^2^ (median, range)	27 (19–47)
GFR ml/min/1.73 m^2^ (median, range)	89 (67–105)
Smoking	
None	4 (36)
Current/former smokers	7 (67)
Pack‐years median (range)	5 (0–40)
Alcohol consumption	
None	0 (0)
Moderate	10 (91)
Heavy	1 (9)

Majority (n = 7) of the population had their treatment protocol modified or treatment discontinued. Four of these treatment modification were related to peripheral sensory neuropathy while others were due to other common chemotherapy induced toxicities.

### 3.2 Feasibility of the NCS measurement

The feasibility of NCS measurements is presented in Figure 1 and Table 2. Baseline NCS measurements were successfully conducted for 6 of 11 patients (54.5%). Encountered technical difficulties in the measurements were nonoccurrence of proper nerve conductivity or no signal. In the case of measurement failure, the equipment was verified for technical errors and new measurements were carried out with increasing the current up to the device maximum (100%). If measurement was still unsuccessful, another study member attempted to re‐measure the nerve conductivity.

**TABLE 2:** Patient demographics stratified by applicability of the point‐of‐care NCS measurement at baseline

Characteristics	NCS applicable *n* (%)	NCS measurement failure *n* (%)
all	6 (100)	5 (100)
Age (median, range)	65.5 (51–70)	73 (52–76)
Gender		
Female	3 (50)	0 (0.0)
Male	3 (50)	5 (100)
ECOG		
0	3 (50)	2 (40)
1	3 (50)	3 (60)
Neurotoxic chemotherapy		
Docetaxel	2 (33)	4 (80)
Oxaliplatin	2 (33)	1 (20)
Vincristine	2 (33)	0 (0)
Comorbidities,		
Hypertension	1 (17)	3 (60)
Diabetes, type 2	0 (0)	2 (40)
Body mass index, kg/m^2^ (median, range)	25.3 (19.0–47.2)	27.2 (22.7–32.0)
GFR, ml/min/1.73 m^2^, (median, range)	90 (83–99)	88 (67–105)
Pack years (median, range)	5 (0–25)	10 (0–40)

The usability of the NCS measurement device was adequate, as all members of the study group conducted successful measurements. Preparation for the measurement, including placement of the electrodes, was estimated to take 5 min per patient.

Next, we carried out an analysis of the baseline characteristics for patients with NCS measurement failure. In the NCS measurement failure group, all the patients were male, they tended to be older, and more frequently had comorbidities, and a history of smoking or were current smokers compared with the successful NCS measurement group (Table 2). However, due to small sample size, none of the differences were statistically significant.

### 3.3 Neuropathy patient‐reported outcomes and adverse events

**FIGURE 2:**
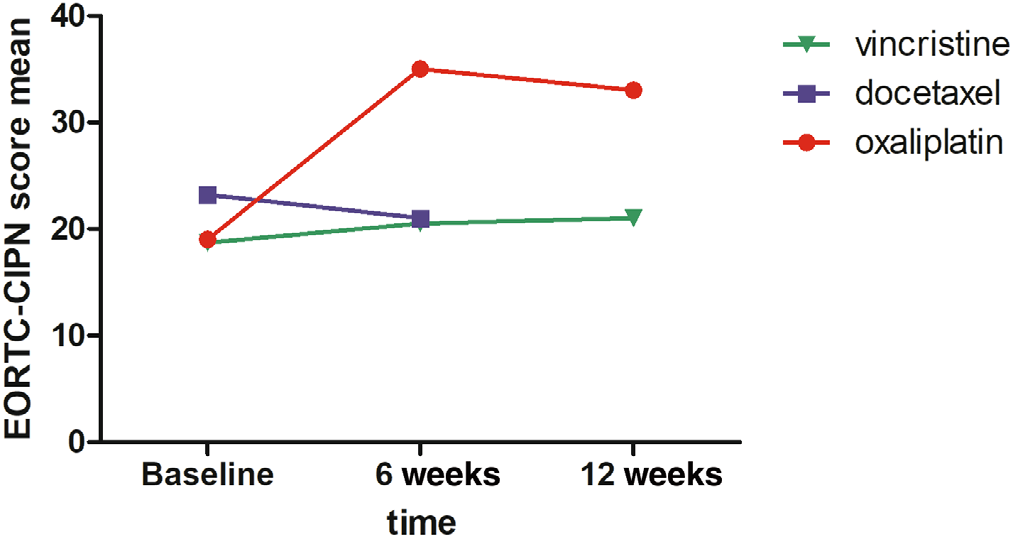
EORTC‐CIPN20 CIPN scores (mean) for the patients according to the used neurotoxic chemotherapy

The patients were analyzed for neuropathy symptoms using validated questionnaires (EORTC‐CIPN20 and NPSI) at baseline and in follow‐up every 6 weeks. Adherence to questionnaires varied since EORTC‐CIPN20 scores were registered for all the study subjects while NPSI questionnaire was found to be non‐feasible since majority of the patients were unable to complete the questionnaire (Table 3). Baseline score of EORTC‐CIPN did not vary between different neurotoxic chemotherapy agents (DOC, OX, and VIN). No change in CIPN20 score were recorded over time which might reflect the dose reductions and discontinuations of the treatments (Figure 2). In the VIN group, only one patient completed CIPN‐20 questionnaires in the follow‐up, and therefore no strong conclusion can be made of these.

**TABLE 3:** The results of the NPSI questionnaire and neuropathy grading

	NPSI score (mean, range)	CTCAE neuropathy grade (grade, *n* of patients)
Vincristine		
Baseline	0 (0–0)	0 (2)
6 weeks	7 (0–14)	1 (1), 2 (1)
12 weeks	0 (0–0)	1 (1), 2 (1)
18 weeks	1 (1)	1 (1), 2 (1)
Docetaxel		
Baseline	0.5 (0–2)	0 (5), 1 (1)
6 weeks	0 (0–0)	0 (2)
12 weeks	n.f.^a^	n.r.^b^
18 weeks	n.f.	n.r.
Oxaliplatin		
Baseline	0 (0–0)	0 (3)
6 weeks	8.5 (0–17)	1 (2)
12 weeks	10.5 (0–21)	1 (2)
18 weeks	10 (7–13)	1 (1), 2 (1)

Abbreviations: CTCAE, common terminology criteria for adverse events; NPSI, neuropathic pain symptom inventory.

^a^ n.f. = not filled in by the patient.

^b^ n.r. = not recorded by the study staff.

Neuropathy was graded by the study staff at 6 weeks intervals co‐occurring with the NCS measurement. Neuropathy grades are presented in Table 3. NCI‐CTCAE neuropathy grades were recorded for 73.3% with the NCS studies. Baseline neuropathy grade was low in all groups. During the course of neurotoxic treatment, neuropathy grades tended to shift to Grades 1–2. However, no strong conclusion can be made about the differences between groups due to the significant lack of follow‐up neuropathy grading in docetaxel group.

### 3.4 NCS measurement results

**FIGURE 3:**
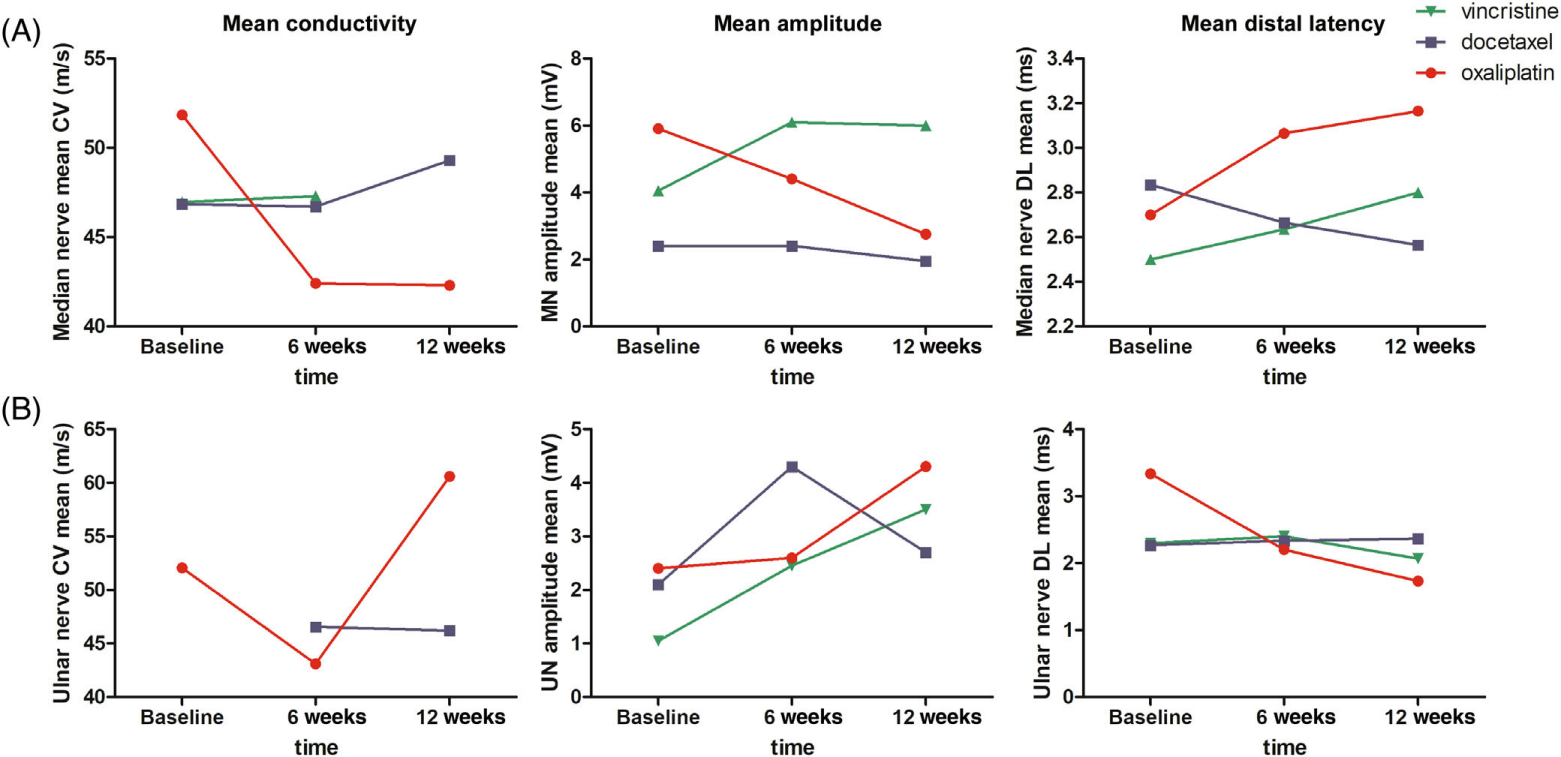
Nerve conductivity measurements for mean conductivity (m/s), mean amplitude (mV). C, mean distal latency (ms) according to the used neurotoxic chemotherapy. (A) measurements for median nerve. (B) measurements for ulnar nerve

The NCS measurement results in chemotherapy groups are presented in Figure 3A,B. All the NCS measurements were done from the dominant upper extremity, with the exception of recurrent measurement failure, where the nondominant side was used. In the OX group, there was a tendency toward decreasing median nerve SNCV and SNAP and increasing median nerve distal latency mean (DL). In the ulnar nerve CV, amplitude, and DL, there were no differences between the groups and there were more measurement failures in the ulnar nerve suggesting lower sensitivity than with median nerve.

## 4 DISCUSSION

To our knowledge, this is the first prospective study of utilizing POCD NCS in analyzing the development of CIPN. Furthermore, the study investigated CIPN scoring methods and their correlation to the POCD measurements. The results suggest that POCD is feasible for some but not all patients receiving neurotoxic chemotherapy.

Mediracer® NCS device has previously been studied and approved for the detection of carpal tunnel syndrome. We speculated that Mediracer® NCS device could also be used in the early detection of CIPN in a rapid, point‐of‐care fashion by the care team. Previous studies have investigated other devices in CIPN detection. A portable POCD for sural nerve conduction (DPNCheck) has shown that peripheral sensory neuropathy grade according to CTCAE grade is associated with a decrease in SNAP in patients with established CIPN.^12^ Another study has investigated established CIPN compared healthy controls with multimodality testing including SNAP, SNCV, LDIFLARE, and QLQ‐CIPN20.^13^ In this study, SNAP and SNCV did not differ while LDIFLARE was significantly reduced in the CIPN group and correlated with QLQ‐CIPN20 results.

In our proof‐of‐concept study, we investigated the feasibility of Mediracer® NCS POCD in detection of CIPN and the correlation to CIPN questionnaires and CTCAE‐based AE assessments. The system setup and required execution time were suitable for point‐of‐care testing. However, the sensitivity of the system was limited in patients with baseline risk factors for peripheral neuropathy, such as age, hypertension, diabetes, and smoking. This suggests that the investigated tool is not feasible for all patients but could be used in the follow‐up of patients without existing peripheral neuropathy risk factors. The measurements on the median nerve had better sensitivity compared to ulnar nerve. In addition to the peripheral sensory neuropathy risk factors, further studies should focus only on the investigating of the median nerve.

The strength of our study is the prospective recruitment of patients receiving three different neurotoxic chemotherapy agents. Compared with the studies by Matsuoka et al. and Sharma et al., our study was more comprehensive in regard to the comorbidities and potential other factors causing peripheral neuropathy. Inter‐rater and intra‐rater reliability of the NCS measurement was ensured by recurrent training sessions with the experienced user of the equipment. In a retrospective pooled analysis of 1401 patients (65 years or older), age and diabetes were independent predictors of CIPN. Smoking was a predisposing factor for long‐term paresthesia among 1402 testicular cancer survivors.^22^, ^23^ These and other potential predisposing factors were considered in our study. The chemotherapy agents in our study are well known for their potential neurotoxicity.^9^

Owing to a feasibility nature of the study, the patient population was limited and further reduced due to the difficulties in the baseline NCS measurements even though the device was used at maximum current setting. Failure of NCS measurement conduction was more frequent in older patients with multiple comorbidities. Furthermore, standard NCS measurement was not used to confirm the findings of the POCD measurements. The NPSI score was frequently not completed in properly, so the score could not be calculated for majority of patients. This may be due to insufficient instructions or difficulties in comprehending the questions.

In conclusion, our pilot study shows the potential of POCD in the early detection of CIPN. Further studies with larger patient populations are needed to fully characterize the clinical utility of the approach. The value of our feasibility study is the preliminary proof‐of‐concept and the definition of the optimal patient population for further clinical testing.

## AUTHOR CONTRIBUTIONS

*Conceptualization*, A.J., S.I., and J.K.; *formal analysis*, A.J., S.I., and J.K.; *investigation*, A.J., S.I., R.M., and J.K.; *Writing – original draft*, A.J., S.I., and J.K.; *methodology*, S.I., K.M., J.K.; *measurements and NCI‐CTCAE grading*, A.J., S.I., J.K., and R.M.; *nerve conductivity measurements*, K.M.; *statistical analysis*, A.J., S.I., and J.K.; *project administration*, J.K.; *resources*, J.K.; *software*, J.K.; *supervision*, J.K.; *validation*, J.K.; *visualization*, J.K. All the authors participated in analysis and interpretation of the data, and drafted, read, and approved the final version of the manuscript.

## FUNDING INFORMATION

This work was supported by the University of Oulu and Oulu University Hospital.

## CONFLICT OF INTEREST

The authors declare no conflict of interest.

## ETHICS STATEMENT

The study was approved by the ethics committee of Northern Osthrobothnia Hospital District (study n:o 48/2019) and registered in an international clinical study registry (NCT04778878). Ethics committee approved written informed consent was sought from all the participants before study procedures. Pseudonymization was carried out before data analysis.

## CONSENT FOR PUBLICATION

All the authors' have read and approved the final version of the manuscript.

## Data Availability

Owing to data protection legislation in Finland, individual‐level data on the study subjects cannot be released.
